# Medical Items and News of Interest to the Physician

**Published:** 1882-10

**Authors:** 


### For the Use of Physicians.

CULLED FROM THE STANDARD MEDICAL JOURNALS OF THE WORLD.

NOTE.—These formulæ are intended only for the regular practitioner of medicine, and should be employed only by him, or under his immediate supervision.

**Herpes Zoster.**

Dr. J. Meredith, of Birmingham, Eng., finds peppermint oil, applied locally, to be capable of rapidly allaying the pain of herpes zoster.

**Intermittent Neuralgia and Cephalalgia.**

℞
Dextro-quiniæ, grs. xxx.
Ammonii chloridi, grs. xx.
M. ft. chart No. vj.

Sig. One powder three times daily "promptly alleviates the sufferings."—*Dr. W. Mathews.*

**Ague.**

Dr. Saunders, of Indore, India, gives nitrite of amyl mixed with an equal amount of oil of coriander, to render it less volatile and mask its taste. Four drops of this are put on a piece of lint, and from it is freely inhaled by a patient in the cold stage. Following the flushing of the face which occurs, perspiration takes place and the cold stage is at an end.

**Erysipelas.**

F. H. Atkins has for eight years made a practice of treating erysipelas by the local application of borax dissolved in glycerine, one drachm to an ounce, which is to be well rubbed in and applied on linen. The result has been to cut the disease short apparently in a few hours. In some cases he used tincture of iron internally, in some not.—*Phil. Med. Times.*

**Relative Mortality Amongst White and Colored Races.**

In his annual report for the past year, the Mayor of Savannah draws attention to the great disparity in the percentage of mortality amongst the white and colored races. The annual rate for 1,000 whites for the year 1880 was 19.85, and for 1,000 colored, 45.47; these rates being calculated on the United States census tables during the same year. In the Mayor's opinion, the disparity is due to a want of observance of the laws of public hygiene, and to neglect, on the part of many of the lower class of colored persons, in ministering to the necessities of the sick.—*Gaillard's Medical Journal.*

**Pruritis.**

Dr. L. Putzel (*Journal of Nervous and Mental Diseases*) reports an obstinate case of pruritis and urticaria which promptly yielded to the fluid extract of jaborandi. Five minims were given thrice daily. The disease always returned when the treatment was discontinued. Jaborandi seems likely to be of value in such conditions.

**Pleasant Preparation of Tincture Ferri Perchloridi.**

℞

Tr. Ferri Perchloridi, ʒ ij.

Pot. Citrat, ʒ j.

Syrup limonis, ℥ iss.

Aquæ ad, ℥ vj.

M. It may also be combined with astringents in this manner.—*Prof. T. D. Reid, Canada Med. and Surg. Journal.*

**Sore Nipples.**

The *Union Medicale du Canada* recommends the following:

℞
Cacao butter, 3 drachms.
Oil of almonds, 1 drachm.
Extract of rathany, 20 grains.

To be applied three times a day, the parts being afterward covered with cotton wadding.

**Styptic Colloid.**

The following will instantly coagulate blood, forming a consistent clot, under which wounds will readily heal:

Collodion, 100 parts.
Carbolic acid, 10 parts.
Tannic acid, 5 parts.
Benzoic acid, 5 parts.

Mix the ingredients in the above order.—*Chemist and Druggist.*

**Alkaline Draughts.**

Where an alkaline drink is indicated the following will be found agreeable:

℞

Bismuthi carbonat } *aa* gr. x.

Magnesiæ carbonat }

Make a powder, to be taken in half a bottle of soda-water three times a day. Or—

℞
Bismuthi subnitrat, gr. xv.
Sodæ bicarbonat, gr. xxij.
Pulv. tragacanthe co, gr. lxij.

Make a powder, to be taken twice or thrice in twenty-four hours in a wineglassful of brandy and water.—*Boston Journal of Chemistry.*

**Collyrium.**

A solution in common use in the Manhattan Eye and Ear Infirmary, of New York, as an application to the eye, is the following. It originated with Dr. C. R. Agnew, and is applied as a spray by means of an atomizer:

℞

Tannin, gr. x.

Biborate of soda, gr. xx.

Glycerin, ʒ ij.

Water, O.ij.

**Vesical Catarrh, Neuralgia and Sciatica.**

The *Druggist Circular* gives the following formula as an excellent mixture in vesical catarrh, neuralgia and sciatica:

℞
Ol. terebinth, ʒ ii.
Ether sulphur, ʒ i.
Mix by shaking violently, and add:
Syrup aurant flor., ℥ i.
Aqua, ℥ iv. M.
Sig.—Teaspoonful every two hours.

**Hemorrhoids.**

Landowski, in *Jour. de Therap.*, suggests hot sitz-baths in bleeding piles, together with enemata of hot water. These not only check the bleeding, but also diminish the size of the turgescent tumors to a marked degree. In ordinary hemorrhoids three sitz-baths per diem may be employed. In bleeding piles the baths should be more frequent, and the enemata should be given as hot as the patient can bear (usually about 104°).

**Fatty Degeneration of the Heart.**

Dr. Kulz administers nitrie of tamyl in those cases, where, on account of fatty degeneration of the heart, it would be imprudent to give digitalis, which by contracting the small vessels might increase arterial tension and cause arrest of the heart. It will always be useful to be able to hold back a remedy like digitalis capable of exciting the vitality of the muscular fibers which are still normal, and await the proper moment of administering it, so as to avoid the sudden deaths unfortunately so frequent. The author employs nitrite of amyl in numerous nervous troubles, painful or spasmodic. He does not recommend it in hysteria or epilepsy, but finds it particularly useful in incipient cardiac paralyses, as it produces peripheral hyperemia, and diminishes arterial tension at the same time it causes the heart to contract.—*Lond. Pract.*

**Neuralgia.**

Dr. David L. Wallace, of Newark, N. J., recommends the following ointment for neuralgia through the columns of the *Medical Record:*

℞

Veratriæ, grs. iv.

Morphiæ, gr, vj.

Tr. Aconite rad., ʒ iss.

Vaselinæ, ℥ j.

M. Sig. To be applied to painful part every fifteen minutes.—*Peoria Medical Monthly.*

**Eczema.**

Dr. H. G. Piffard (*Medical Record*) is inclined to believe that viola tricolor is a specific in eczema, in the same sense that quinine is a specific for ague. He uses fluid extract; the dose being, in adults, from five to ten minims in acute eczema, and from half a drachm to two drachms in subacute and chronic eczema. The drug should be taken in water once, twice or thrice a day, always on an empty stomach, and, when possible, half an hour before meals.—*Chicago Med. Review, May*, 1882.

**Diabetes.**

Professor Da Costa reports six cases of diabetes insipidus treated with ergot, with five recoveries. The remedy is given in the form of fluid extract and not less than three fluid drachms per day, and this amount should be gradually increased to double its quantity. It may be well to follow the ergot, after the amount of urine has been reduced to the normal, with strychnia. In other cases, where the ergot must be continued for some time, codliver oil is a good adjunct.—*Medical News.*

**Antipruritic.**

Dr. Bulkley has directed attention to a very important point which is often a source of great anxiety to the practitioner, viz., the difficulty in relieving persistent and wearing itching in skin affections. He points out that the drugs we certainly rely on, viz.: Opium, morphia, chloral, bromide of potassium, aconite, and carbolic acid, when administered internally, often fail to stop the unconscious scratching, and he was led from the known effects of gelsemium to try that drug. In certain cases he has found it decidedly efficacious. He begins with ten drops of the tincture, and, if in half an hour there is no relief he gives twelve or fifteen drops, and so on, until one or two drachms have been reached in two hours.—*Buffalo M. and S. Journal.*

**Alopæcia and Seborrhœa.**

The oil of ergot is recommended by Dr. J. V. Shoemaker as a remedy for alopæcia and seborrhœa. It has been in use several years and finds much favor with the profession. The favorite formula for its application is as follows:

℞

Oleate of mercury, ℥ ss.

Olei ergotae, ℥ iiss.

Ol. rosae, gtt ii. M.

Sig.—Apply to the parts.

**Calcium Sulphide in Small-Pox.**

Dr. R. H. Cowan, of Richmond, Va., writes: "Since reading in a recent number of the *Record* a report read before the New York County Society on the use of calcium sulphide as an antisuppurative, the administration of this drug in small-pox has strongly suggested itself to my mind. As our means of combating this fearful disease are so limited, and the results of treatment so poor, it seems to me that calcium sulphide would be worth a trial." —*Med. Record.*

**Spasmotic Croup.**

Dr. G. L. Gray, in *Miss. Val. Med. Monthly*, states that he has used cotton seed successfully. Take a handful of seed, bruise them, boil in a quart of water for a few minutes, let it stand a short time, strain, sweeten, and, when cool enough, give the patient all it will drink, or if necessary, pour it into the child. The relief is generally prompt and sometimes without emesis. If persisted in, it produces free emesis. Dr. Gray also states that he has used the remedy with benefit in two cases of humoral asthma. —*Va. Medical Monthly.*

**Bright's Disease.**

Fuchsine has been used extensively by Dr. Renzi, of Geneva, given in pill form, .025 gram (5-12 grain) twice a day. There was at once a noted diminution of the albumen and dropsy. The urine was colored for some days. No results followed in one case. Similar experience was had by Brochut, of Paris. In every case albumen disappeared rapidly and entirely, the treatment generally lasting from four to six months. Dr. James Sawyer has used it mostly in cases of contracted kidney, with good results and no untoward physiological effect. It colors the mucous membranes of the digestive organs a deep red and also the plasma of the blood, due to its presence, not to blood change.—*Canada Medical and Surgical Journal.*

**Diarrhœa.**

Prof. William Thomson, of the University of the City of New York, recommends the following as a remedy for diarrhœa:

℞
Plumbi acetatis, gr. xvi.
Pulv. camphoræ, gr. xij.
Pulv. opii, gr. iij.
Bismuth subcarb, gr. xij.
Ext. gentian, q. s.

Make into 12 pills. Dose, one pill every hour to three hours, according to severity of disease.

**Acute Metritis.**

Dr. J. Warren, Boston, recommends the following internal medication for relieving the engorged state of acute metritis:

℞

Chloral hydrat., ʒ iii.

Chloral croton, gr. xxx.

Liq. opii. com., ʒ vi.

Glycerinæ, ℥ ii.

Syr. tolu., ℥ i.

M. Teaspoonful every hour until ease from pain; and sleep is produced.—*Kansas Med. Index.*

**Prurigo and Eczema.**

In a paper read before the Michigan Medical Society, Dr. D. W. C. Wade says that the subjoined formulæ can not, in his opinion, be profitably replaced by any thing else to be found in the literature of the subject. In many other of the skin affections there are reasons for expecting results no less satisfactory.

PRURIGO OINTMENT.

Washed sulphur, 30 grains.
Carbonate of ammonia, 30 grains.
Petroleum ointment, 1 ounce.
Perfume, sufficient.

Mix. Apply at night, and wash with soap in the morning.

ECZEMA LOTION.

Powdered iodoform, 30 grains.
Subnitrate of bismuth, 60 grains.
Hydrate of chloral, 15 grains.
Glycerine, 1 drachm.
Oil of rose geranium, 15 drops.
Water, 3 fluid ounces.

Mix. Shake, and apply one to three times daily.

This formula, in varying the proportion of water, or substituting one ounce of tincture of opium, constitutes a favorite local panacea for the many affections of the skin and mucous membranes.

**Tonsillitis.**

Seven cases of tonsillitis cured by the use of bicarbonate of soda are reported by Dr. Armangue in *Revue de Therapeutique.* This method was introduced by Dr. Gine, Professor of Clinical Surgery. What is still more remarkable, these cases are said to have all been cured in less than twenty-four hours. The mode of employing this agent is either by insufflation, or directly applied by the finger of the patient. The alleviation is almost always immediate, and rarely delayed more than twenty-four hours. The frequent application of bicarbonate of soda is said to reduce the size of the gland in hypertrophy and thus render excision of the tonsils unnecessary.—*Therapeutical Gazette.*

---

**Gonorrhœa Suppositories.**

Dr. D. W. C. Wade considers this affection to be a fermentative disease. He proposes the following plan of treatment which has a very strong support from a clinical standpoint. Take of:

℞

Powdered iodoform, 2 drachms.
Subnitrate of bismuth, 2 drachms.
Hydrate of chloral, 15 grains.
Morphia, 5 grains.
Oil of rose geranium, 20 drops.
Cacao butter, 1 ounce.

Mix and divide into twenty-four suppositories one-eighth of an inch in diameter. Directions, one to be pushed into the urethra three times daily.

---

**Asthma.**

The grindelia robusta, an asteroidea growing on the slopes of the west coast of America, which is used in those regions as the safest remedy against asthma, has of late attracted the attention of physicians in an increased degree. In favor of this new remedial plant with reference to the therapeutic effects, it is claimed that the plant is not numbered among the narcotics, as the solaneas (hyoscyamus, stramonium, etc.), which have been used heretofore, but it comes from a family which altogether furnishes us "hervica." The grindelia robusta is, according to experience, the most effective species. The resin is an essential part of its effectiveness.

Quantities of this resin, taken in proportion to the quantity of tobacco used, were employed to impregnate well nitred tobacco, and from this cigarettes were formed, which, after long observations during several months, have always satisfactorily benefited those asthmatics which have used them. The use of these cigarettes, which are easily lit, and either smoked, or the smoke of which is fanned towards the patient, is recommended by the author for every patient, even for non-smokers (ladies), as the cigarette once lit, on account of the quantity of saltpeter with which it is impregnated, will continue to glow and develop smoke.—Von Bombelon (Bergen auf Rugen), *Deutsche Med. Wochenschrift.*

---

**Death from Undigested Bismuth.**

An Italian medical paper relates that a woman died of gastric cancer in the hospital at Modena, on March 2, 1881, having been a patient since January 5th preceding. The postmortem examination brought to light a singular and noteworthy fact. The stomach, which was of normal size, was divided along its greater curvature, and was then seen to be filled by a pultaceous gray, semi-solid mass, forming a cast of the cavity. Upon examination it was found to be made up of half a kilogram of oxide of bismuth agglutinated with mucus. The neoplasm was situated three fingers' breadths from the pylorus, and proved to be cancerous. As it was established that the patient had taken no bismuth while in the hospital, it must be admitted that she had carried the mass in her stomach for at least two months.

---

**Delphinium Ajacis.**

Dr. Benvenuti has published the results of his researches upon the virtues of *Delphinium ajacis* (rocket larkspur). A cold acetic-acid infusion of the flowers in many cases of phtheiriasis pubis was very efficient in destroying parasites and ova. In several examples of intractable ulcerating bubo, lint soaked with the above infusion brought about speedy cicatrization. An aqueous infusion of the dried flowers brought about similar results in instances of phagedenic ulcers, virulent wounds, and adenitis seen in prostitutes. From his experiments the author draws the following conclusions: The flowers of the delphinium possess an insecticide action. They are to be preferred to other remedies of similar action on account of cheapness and absence of smell. They have a marked anæsthetic action, are excitant, rubefacient, astringent, and antizymotic. The author thinks this remedy has many points of resemblance to carbolic acid and iodoform.—*London Practitioner.*

**Sedative Emmenagogue.**

For a day or two antecedent to the actual commencement of the catamenial flux, women not infrequently suffer acute pain in the pelvic region, doubtless due to hyperæmia and hyperæsthesia of the reproductive belongings. To obviate this I have found no treatment give such satisfactory results as the following:

℞

Codeiæ sulphatis, gr. j.
Chloral hydratis.
Ammonii bromidi, aa gr. xx.
Aquæ camphoræ, ℥ j.

M. For one dose. S. Take at bedtime.

A repetition of the dose at that period is rarely necessary. In some cases a warm sitz-bath of fifteen minutes' duration before retiring, is a valuable adjuvant.—*Western Med. Rep.*—*Virginia Med. Monthly.*

---

**Diphtheria.**

Dr. Lolli, of Trieste, uses exclusively the following mixture in the treatment of diphtheria, and in sixty cases the mortality was less than two per cent., the malady having a duration of but eight or ten days, and being but rarely propagated to the mucous membrane of the respiratory organs:—

℞

Ferri sesquichlorid., gr. xv gr. xlv.
Ac. carbolic. pur. gr. xv. gr. xlv.
Mel. Rosæ, ℥ j.
Aquæ calcis, ℥ xv. M.

The throat is swabbed with this mixture every half hour, adults using it as a gargle, and it is, besides, to be taken in tablespoon doses, diluted, every second hour. Of course tonics and very nourishing food form most important adjuncts to the treatment.

---

**Nasal Catarrh.**

Dr. N. Falliott, writing to the *British Medical Journal*, states that coryza or nasal catarrh may be cured in a few hours if taken at the onset, or at most twelve hours afterward, by the inhalation of a spray of sulphate quinine. The solution used is made by dissolving four grains of quinine in an ounce of water, with just enough of sufficiently dilute sulphuric acid to dissolve it, and scenting with any agreeable perfume. The solution is injected up the nostrils in the form of spray with an ordinary hand ball spray producer in such a way that the quinine can be tasted at the back of the mouth. This is done every hour or oftener, according to the urgency of the symptoms. He states that this remedy has been tried with success in hay fever, and that if nasal catarrh is of parasitic origin, as he strongly suspects, the action of quinine (as an antiseptic ?) is at once apparent. It might be added, that even supposing catarrh to be the result of sudden change of temperature, the action of quinine in contracting the superficial capillaries would be quite as obvious. It is somewhat surprising that this property of quinine does not appear to have been tried for chilblains in the itching state, when the capillary vessels are dilated.—*New Remedies—Mattison Monthly Review.*

---

**Micro-Organisms.**

N. P. Wassilieff, after noting the high estimation in which calomel has been always held in disordered conditions of the bowels, especially in children, points out the absence of any experiments to show or explain the cause of its influence, with one or two exceptions. Voit, indeed, as long ago as 1837, observed that albumen and blood, mingled with calomel, were capable of being kept for days without any indication of putrefaction, and Hoppe-Seyler made some similar observations; but besides these, few, if any, researches have been made on its action. In M. Wassileff's experiments, calomel was added to the fluid obtained by acting on albumen with pancreatic juice; and he satisfied himself that the albumen-digesting ferment of both these fluids was not injured by calomel—peptones in the one instance, and leucin and tyrosin in the other, appearing as usual—but that the presence of this substance prevented the formation of the secondary products, such as indol and phenol. Neither hydrogen nor hydrogen sulphide formed in the fluids containing calomel, whilst they were abundantly produced in the others. In like manner, the author experimented on the effect of calomel on the fat-digesting and the amylolytic ferments of the pancreas, and found that it had no modifying influence upon them, but that it arrested the changes which followed their completion, entirely preventing, for example, the butyric acid fermentation and putrefactive processes. He hence arrives at the conclusion that calomel acts different on the formed or organized and the unformed or unorganized ferments, permitting the action of the former to proceed unchecked, but completely preventing the action of the latter.—*Lancet.*

**Rabies.**

At a recent meeting of the Societe Med. des Hopitaux, of Paris, M. Gingeot remarked that in a case of rabies he had employed the hoang-nan, said to be much employed among Eastern nations. He did not observe any beneficial effects from its use, but it was not given until a late period of the malady. He considers that the medicament is best administered hypodermically, using a watery solution of the alcoholic extract, as the pills are swallowed with difficulty and frequently rejected by vomiting.

M. Dujardin-Beaumetz considers the Russian method of treatment the best of those at present known. The patient is placed in an overheated room and garlic or sulphide of allyl is administered. Six months since he had three persons under his care, who had been bitten by a dog undoubtedly mad; they were submitted to this form of treatment in all its details, and escaped without presenting any symptoms of rabies. Valdivine, which has been vaunted in this affection, has proved of no value in his hands.

M. Sevestre said that an attenuation of the symptoms was obtained in one case by the administration of pilocarpine, but the patient finally succumbed.

---

**Alkalies on the Urine.**

In an interesting work on the subject of the "Morbid Conditions of the Urine dependent on Derangements of Digestion," Dr. Ralfe has brought forward additional evidence to prove the difference in the effect of alkalies determined by the period of their administration. It is not sufficiently known that the changes in the urinary secretion effected by alkalies differ widely when administered before meals and during digestion. In 1850, Dr. Bence-Jones—a physician much in advance of his time, by the way—demonstrated that large doses of carbonate of ammonia greatly increased the acidity of the urine on the following day. Four years afterward, Beneke made the same observation for carbonate of soda, and subsequently Parkes demonstrated the same result from the administration of bicarbonate of potash. This fact might, indeed, have been supposed, *a priori*, from the laws of diffusion. When a quantity of alkali is administered before meals, the acid-forming constituents of the blood must diffuse into the gastric juice. An immense quantity of acid material must then, during digestion and at its close, diffuse again into the blood from the stomach, and be eliminated some hours hence by the kidneys.

Far different is the result when any considerable quantity of alkali is administered during digestion. The acid already produced is neutralized, and, for some hours subsequently, a more or less alkaline fluid diffuses into the blood, and is eliminated by the kidneys. The influence which these observations ought to have on the time for the administration of alkalies is obvious. That they are new, is not to be affirmed in view of the brief history given above, but that they are generally known, can not be alleged.

Applying the same principles to the administration of acids, we obtain correct guidance. Given before meals, they check excessive production of acid gastric juice; given during digestion, they add to the acidity of the stomach, and, consequently, of the blood. That these principles are generally applied, our observation does not permit us to conclude. We have reason to believe that they have been fully described in lectures on therapeutics, delivered during successive years in this city.—*Medical News.*

---

**Aural Vertigo.**

In a paper read by Dr. C. H. Burnett before the Philadelphia County Medical Society on this subject, the following conclusions are drawn:

1. That there are two sets of fibres in the auditory nerve, viz.: the sensory and the motor.

2. That the motor filaments are connected on one side with the cerebellum by means of the inferior peduncles, and on the other side with the nerve filaments sent to the ampullæ of the semi-circular canals.

3. That irritation of these ampullar nerves may be conveyed from either of the three parts of the auditory apparatus, or from the auditory nerve itself, in the mechanical form of pressure, and that this irritation may be further conveyed to the cerebellum and cause vertigo; so that it logically follows that this reflex cerebellar phenomenon as produced by aural irritation should receive the general denomination of *aural vertigo*, and that Meniere's disease is only a form of aural vertigo. Hence the latter name, unless used after accurate diagnosis of a disease originating in the labyrinth, *i. e.*, in the semi-circular canals, will create confusion.—*Philadelphia Med. Times.*

**Antisuppurative.**

Dr. Andrew H. Smith, Chairman of the Committee on Restoratives of the Therapeutical Society of New York, furnishes to the *New York Medical Journal and Obstetrical Review* a report of the committee on the use of sulphide of calcium for the purpose of preventing or diminishing suppuration. After giving the experience of several members of the society, Dr. Smith concludes his report as follows: Judging from this limited number of cases, it would seem that we are warranted in concluding that in many cases of suppurative affections, ranging from the small pustules of acne to extensive suppurating surfaces, an appreciable, and often a very marked, benefit is derived from the use of the calcium sulphide, suppuration which would otherwise take place being averted, or the quantity and duration of an existing discharge being lessened. At the same time its action is not uniform; and in many apparently favorable cases it will fail entirely. The drug is somewhat prone to irritate the stomach, and this circumstance affords an indication for small doses frequently repeated instead of larger ones at long intervals. One-tenth of a grain every two hours in acute cases will generally secure the full therapeutical action of the drug, but larger doses may sometimes be required, and some patients will bear well a grain three or four times a day. Even in small doses the sulphide will occasionally produce headache, and the patient is usually more or less annoyed by eructation of sulphuretted hydrogen.

---

**Constipation in Infants.**

The following are some of the remedies found useful by Dr. D. H. Cullimore (*London Lancet*): 1. A pellet of butter and brown sugar or treacle every morning fasting, or a little raspberry jam. 2. The morning insertion into the rectum of a conical piece of white curd soap about two inches and a half long. It must be first dipped in warm water, held *in situ* for five minutes, and withdrawn. 3. Daily friction over the body, from the right iliac region along the course of the gut, with a little salad oil. In India I have used cocoanut oil advantageously. Cod liver oil is very useful when its smell is not objected to. *En passant*, I may say that I have at present under my care a girl of fifteen, who, for a couple of months, has suffered from obstinate constipation. She has lately had typhoid. Both mild and strong purgatives were ineffectual, and it has now yielded to cod liver oil friction. Assiduous friction, without any unguent, is often equally useful. Patience, however, is necessary. A teaspoonful of fluid magnesia in the food is a good plan. Tomato jelly is sometimes used in India with benefit. Whatever plan may be adopted, it is well to supplement it with the internal administration of half a drop of tincture of nux vomica three times a day; a quarter of a drop is sometimes sufficient. Minute doses of sulphur also answer well.—*Louisville Medical News.*

---

**Malaria.**

Dr. R. B. Morrison reports in the *Maryland Medical Journal* two hundred and fifty cases of malaria treated with tincture of iodine, in fifteen-minim doses, largely diluted, a quarter-hour before meals. His cases show an even more favorable result than where cinchonidia is employed, and he states that iodine is now the routine treatment in all cases of acute malaria coming to the dispensary. Similar observations are reported in the *Indian Medical Gazette* for January by Buboo Brojendry Nath Bamergee, at the meeting of the Calcutta Medical Society. He states that since 1878 he has made large use of this method of treatment, and in 1879 published in the *Calcutta Medical News* that he had cured ninety per cent. of five hundred cases. Since then, however, he has been led to distrust the reliability of his results, and believes that many of his earlier trials were made in cases which recover spontaneously, while others of these cases last, even under no treatment, only one day. Under such circumstances, any drug will appear to act like magic. His present belief is that iodine will prove serviceable in about half the cases. The same author also reports cases in which burnt alum appeared to be of service.

---

**Scarlatina.**

Dr. J. A. Octerlony (*American Journal of the Medical Sciences*, July, 1882), basing his opinion upon the careful study of fifty-eight cases of scarlatina occurring under his own observation, advocates the theory of Eklund, of Stockholm, whose observations as to the parasitic nature of this disease he has repeatedly confirmed. In the urine of scarlatinous patients there is constantly present an immense number of peculiar cellular bodies which have received the name plax scindens. They consist of sporodial cells, flat, oval, or round, and either colorless or yellowish white; they have a distinct cellwall, and a nucleus of a clear brownish color. Sometimes the nucleus contains a very minute nucleolus. As seen floating about in the fluid examined, they often exhibit rotary or screwing or sea-saw movements. It has been further observed that these little bodies multiply by division of the nucleolus, then the nucleus divides, lastly the cell itself undergoes division; mycelium filaments never develop from these cells, nor do they arrange themselves in beads or in the zooglœa form. These bodies are always found in the blood and urine of scarlatinous patients.—*Chicago Medical Review.*

---

**Embalming.**

The process of embalming is as follows, and is called the "Brunelli process." 1. The circulatory system is cleansed by washing with cold water till it issues quite clear from the body. This may occupy from two to five hours. 2. Alcohol is injected, so as to abstract as much water as possible. This occupies about a quarter of an hour. 3. Ether is then injected to abstract the fatty matter. This occupies from two to ten hours. 4. A strong solution of tannin is then injected. This occupies for imbibition two to ten hours. 5. The body is then dried in a current of warm air passed over heated chloride of calcium. This may occupy two to five hours. The body is then perfectly preserved, and resists decay. The Italians exhibit specimens which are as hard as stone, retain the shape perfectly, and are equal to the best wax models. It will be observed in this process that those substances most prone to decay are removed, and the remaining portions are converted by the tannin into a substance resembling leather.

---

**Hydrocele.**

On the 20th of April, 1864, without drawing off the water of the tumor, I injected into a hydrocele, with a hypodermic syringe, about thirty drops of strong compound tincture of iodine, thinking that the dilution of the iodine in the fluid of the hydrocele would stimulate the sac sufficiently, and that the next day the water could be drawn off and the surfaces of the vaginal sac be thus allowed to come in contact. To my surprise, the next day the hydrocele was not half the size; the fluid had been absorbed. Instead, therefore, of drawing off the water, on the 23d I repeated the iodine injection, and on the 26th, though the swelling had been still more reduced, I again threw in the iodine. On the 30th the fluid had disappeared through the vaginal coverings and the testicle was thicker and hung down lower than on the side not implicated. He wore a suspensory bag from the third day after the first injection, and I directed him to continue to wear this, making it a little tighter than he had been wearing it. From the first injection this patient experienced no pain or inconvenience and did not lose an hour from his work. He had no return of his disease six months after the operation. The cure therefore was complete. Encouraged by the success of this operation, I have treated successfully eleven other cases, and my friend, Dr. I. S. Mitchell, at my suggestion, has treated five cases with like success. I have not tried this in very old hydroceles.—*J. L. Ogler, Charleston, S. C., in American Med. Journal.*

---

**Eczema of the Genitalia, Pruritus and Leucorrhœa.**

In cases of eczema, in which glyceroles and unguents have failed, the following formula has been successful:

℞
Chlorate of potassium, 30 grammes.
Wine of opium, 50 grammes.
Pure water, one quart.

Applied to the parts by linen compresses covered with oiled silk. If there is much inflammation, precede this with warm hip baths and cataplasms sprinkled with powdered carbonate of lime. In obstinate pruritus, associated with leucorrhœa, a tablespoonful of mixture of equal parts of tincture of iodine and iodide of potassium, in a quart of warm tar water (tar water holding the iodine in solution) used daily, night and morning, removes the pruitus and ameliorates the leucorrhœa. In fetid leucorrhœa two or three tablespoonfuls (in a quart of warm water morning and evening, as an injection) of the following formula, will be found useful:

℞
Chlorate of potassium, 12 grammes.
Wine of Opium, 10 grammes.
Tar water, 300 grammes.

Or,
℞
White vinegar (or wine), 300 grammes.
Tinct. eucalyptus, 45 grammes.
Acid salicylic, 1 gramme.
Salicylate of Soda, 20 grammes.

One to five teaspoonfuls in a quart of warm water as an injection two or three times a day. —*Review of Gynecology, Obstetric Gazette.*

